# The characteristics of blood glucose fluctuations in patients with fulminant type 1 diabetes mellitus in the stable stage

**DOI:** 10.20945/2359-3997000000082

**Published:** 2018-10-01

**Authors:** Jie Wang, Bing-Li Liu, Zheng Li, Hui-Qin Li, Rui Sun, Yun Hu, Kok-Onn Lee, Lei Ye, Xiao-Fei Su, Jian-Hua Ma

**Affiliations:** 1 Nanjing Medical University Nanjing Medical University Nanjing First Hospital Department of Endocrinology Nanjing China Department of Endocrinology, Nanjing First Hospital, Nanjing Medical University, Nanjing, China; 2 National University of Singapore Department of Medicine Division of Endocrinology Singapore Division of Endocrinology, Department of Medicine, National University of Singapore, Singapore; 3 National Heart Research Institute Singapore National Heart Centre Singapore Singapore National Heart Research Institute Singapore, National Heart Centre Singapore, Singapore

**Keywords:** Fulminant type 1 diabetes, type 1 diabetes, continuous glucose monitoring system (CGMS)

## Abstract

**Objective:**

The aim was to characterize blood glucose fluctuations in patients with fulminant type 1 diabetes (FT1DM) at the stable stage using continuous blood glucose monitoring systems (CGMSs).

**Subjects and methods:**

Ten patients with FT1DM and 20 patients with classic type 1 diabetes mellitus (T1DM) (the control group) were monitored using CGMSs for 72 hours.

**Results:**

The CGMS data showed that the mean blood glucose (MBG), the standard deviation of the blood glucose (SDBG), the mean amplitude glycemic excursions (MAGE), the blood glucose areas and the percentages of blood glucose levels below 13.9 mmol/L were similar between the two groups. However, the percentage of blood glucose levels below 3.9 mmol/L was significantly higher in the FT1DM group compared to the T1DM group (p < 0.05). The minimum (Min) blood glucose level in the FT1DM group was significantly lower than that of the T1DM group (p < 0.05). Patients with FT1DM had severe dysfunction of the islet beta cells and alpha cells compared to patients with T1DM, as indicated by lower C-peptide values and higher glucagon/C-peptide values.

**Conclusion:**

In conclusion, patients with FT1DM at the stable stage were more prone to hypoglycemic episodes as recorded by CGMSs, and they had a greater association with severe dysfunction of both the beta and alpha islet cells compared to patients with T1DM.

## INTRODUCTION

Type 1 diabetes mellitus (T1DM) is characterized by insulin deficiency and the destruction of pancreatic beta cells. It is mainly classified into classic T1DM and fulminant type 1 diabetes mellitus (FT1DM). FT1DM is a special subtype of type 1 diabetes first proposed by Imagawa and cols. in 2000 (1). FT1DM is characterized by an abrupt onset, dangerous conditions, ketosis or ketoacidosis at the time of diagnosis, high blood glucose levels, lower glycosylated hemoglobin (HbA1c) levels, serious destruction of pancreatic beta cells, and negative islet-related autoantibodies, such as insulin antibodies, anti-GAD antibodies (GADAb), and insulin autoantibodies (IAA). In addition, most patients with FT1DM report having had symptoms of influenza or gastrointestinal symptoms prior to onset (2). Recently, reports on FT1DM have increased, especially in East Asia (2-6). However, detailed characteristics of glucose fluctuations in patients with FT1DM at the stable stage are unclear.

The current study aimed to utilize CGMSs to characterize blood glucose fluctuations in patients with FT1DM at the stable stage.

## SUBJECTS AND METHODS

### Subjects

This was a retrospective study. We identified 10 patients with FT1DM at the stable stage and 20 patients with classic T1DM as control patients from Jan. 2012 to Dec. 2015 at Nanjing First Hospital, Nanjing, Jiangsu, China. The FT1DM group included 6 female and 4 male patients, and their mean history of T1DM was 7.20 ± 5.83 years. All of the patients with FT1DM met the diagnostic criteria proposed by Imagawa: 1) ketosis or ketoacidosis that occurred in a high glucose state, usually less than 1 week; 2) plasma glucose levels > 16 mmol/L (> 288 mg/dL) and HbA1c levels < 8.7% in newly diagnosed patients; 3) excretion of urinary C-peptide < 10 ug/dL with a fasting plasma C-peptide (C-P) < 0.1 nmol/L (0.3 ug/L) and stimulation plasma C-peptide (after a meal or glucagon injection) < 0.17 nmol/L (0.5 µg/L) (7). The T1DM group included 8 female and 12 male patients, and their mean history of T1DM was 9.60 ± 8.40 years. The diagnosis of T1DM was based on the criteria for classical T1DM introduced by the WHO in 1999 (8). The protocol and informed consent documents were approved by the Institutional Ethics Committee at Nanjing First Hospital. All of the patients gave written informed consent.

### Methods

Demographic information about the patients was collected, including each patient's age, history of diabetes, BMI, waist circumference, hip circumference, waist-hip ratio, and insulin dose.

Continuous glucose data were obtained using the Medtronic Minimed CGMS Gold (Medtronic Incorporated, Northridge, USA) for at least 3 days after being admitted to the hospital for 2 days. The CGMS induction probe detected the concentration of abdominal subcutaneous interstitial fluid glucose in the subjects. It was connected to a recorder via a cable so that the glucose concentrations could be obtained and recorded every 5 minutes (min). A total of 288 glucose readings were recorded automatically every day for three days, and the range of blood glucose levels recorded was 2.2-22.2 mmol/L (39.6-399.6 mg/dL). Peripheral blood glucose levels were measured at least 4 times per day during the monitoring period to calibrate the CGMS results. The data obtained from the CGMSs included the following parameters: the 24-hour (hr) mean blood glucose level (MBG), the standard deviation of the MBG level (SDBG), the 24-hr mean amplitude of glycemic excursions (MAGE), the percentage time durations (%) of hyperglycemia (glucose > 13.3 mmol/L) (> 239.4 mg/dL) and hypoglycemia (glucose < 3.9 mmol/L) (< 70.2 mg/dL), the maximum blood glucose level (Max) and the minimum blood glucose level (Min).

The clinical and biochemical parameters included the following: fasting C-peptide (C-P0), 2-hr postprandial C-peptide (C-P2hr), fasting glucagon (Glu0), 2-hr postprandial glucagon (Glu2hr), HbA1c, anti-GAD or insulin antibodies, blood routine, biochemical indexes, and calculations for fasting glucagon/fasting C-peptide (Glu0/C-P0) and 2-hr postprandial glucagon/2-hr postprandial C-peptide (Glu2hr/C-P2hr). Plasma glucose levels were measured using the glucose oxidase method. C-peptide was measured using a chemiluminescent immunometric assay on the Modular Analytics E170 (Roche^®^ Diagnostics GmbH, Mannheim, Germany). Glucagon levels were determined using a quantitative radioimmunoprecipitation assay kit (Beijing North Institute of Biological Technology, China). HbA1c levels were measured using a high-performance liquid chromatography (HPLC) assay (Bio-Rad Laboratories, Inc. CA, USA). The breakfast for the patients’ OGTT was 250 ml milk, 0.1 kg steamed bread meal, and an egg.

### Statistical analysis

For normally distributed data, the means ± standard deviations (SDs) of the two groups were compared using t-tests. For data that were nonnormally distributed, the medians and interquartile ranges were compared using nonparametric Mann-Whitney U tests. The rates between the two groups were compared using the chi-squared test. Significance was defined as p < 0.05.

## RESULTS

### Patient demographics

There were no significant differences in the genders and histories of diabetes between the two groups. Ages, BMIs, and waist-to-hip ratios in the FT1DM group were higher than those in the T1DM group (p < 0.05 for all); premeal insulin doses (unit/kg/day) were significantly higher in the FT1DM group compared to the T1DM group (p < 0.05). Daily doses of insulin (unit/kg/day) and basal doses of insulin (unit/kg/day) were similar between the two groups (p > 0.05) (Table 1).

**Table 1:** Clinical and biochemical characteristics

Groups	FT1DM (n = 10)	T1DM (n = 20)	t/z/χ^2^	P
Gender (%)	4/6	12/8	1.071	0.442
Age (years)	43 (37.5, 54)	35 (26.75, 38.75)	-2.312	0.021*
History (years)	6 (3.25, 10.5)	9.5 (2.25,17)	-0.596	0.551
Body mass index (kg/m^2^)	23.01 ± 2.64	20.98 ± 2.01	2.348	0.026*
Wasit to hip ratio (%)	0.98 ± 0.04	0.93 ± 0.06	2.454	0.021*
The basal dosages of insulin (u/kg/day)	0.24 ± 0.08	0.27 ± 0.15	-0.582	0.565
The pre-meal dosages of insulin (u/kg/day)	0.31 (0.26, 0.61)	0.24 (0.17, 0.31)	-2.268	0.023*
The daily dosages of Insulin (u/kg/day)	0.63 ± 0.20	0.52 ± 0.18	1.657	0.109
Fasting C-peptide (µg/L)	0.01 (0.01, 0.04)	0.07 (0.01, 0.20)	-2.227	0.026*
2-hr postprandial C-peptide (µg/L)	0.01 (0.01, 0.04)	0.11 (0.02, 0.52)	-2.778	0.005**
Fasting glucagon (ng/L)	156.06 ± 43.86	136.19 ± 28.03	1.512	0.142
2-hr postprandial glucagon (ng/L)	208.74 (166.39, 271.73)	165.31 (144.93, 200.39)	-2.202	0.028*
Fasting glucagon/Fasting C-peptide (%)	13271.00 (5193.94, 18294.25)	2227.86 (627.63, 13705.75)	-2.024	0.043*
2-hr postprandial glucagon/2-hr postprandial C-peptide (%)	17595.50 (9020.94, 26907.00)	1339.37 (291.14, 7466.25)	-3.08	0.002**
Glycosylated hemoglobin (%)	8.03 ± 1.31	9.70 ± 2.65	-1.864	0.073*
Anti-GAD antibodies (%)	5.48 (4.67, 7.21)	13.40 (9.36, 23.03)	-3.696	0.000**
Anti-Insulin antibodies (%)	3.37 (2.86, 8.26)	6.09 (2.95, 11.62)	-1.16	0.246
White blood cells (*10^9^/L)	6.02 ± 3.51	6.88 ± 2.83	-0.728	0.473
Red blood cells (*10^12^/L)	4.12 ± 0.44	4.42 ± 0.49	-1.632	0.114
Platelet count (*10^9^/L)	156.8 ± 56.13	196.05 ± 63.82	-1.649	0.110
Haemoglobin (g/L)	131 ± 9.24	134.85 ± 18.51	-0.617	0.543
Alanine aminotransferase (U/L)	25.00 (16.50, 38.75)	19.00 (11.00, 24.75)	-1.652	0.099
Albumin (g/L)	41.86 ± 9.67	42.63 ± 3.59	-0.317	0.754
Globulin (g/L)	25.32 ± 3.76	25.49 ± 4.77	-0.098	0.922
Potassium (mmol/L)	4.05 (3.71, 4.27)	4.42 (3.79, 4.74)	-1.651	0.099
Sodium (mmol/L)	141.05 ± 4.87	138.94 ± 4	1.269	0.215
Chloride (mmol/L)	102.97 ± 5.59	102.15 ± 4.08	0.461	0.648
Cholesterol (mmol/L)	4.66 ± 0.77	5.26 ± 0.94	-1.736	0.094
Triglyceride (mmol/L)	1.12 ± 0.78	1.03 ± 0.71	0.315	0.755
HDL-cholesterol (mmol/L)	1.5 ± 0.51	1.77 ± 0.53	-1.344	0.190
Glutamyl transpeptidase (U/L)	18.5 (12.00, 27.75)	15.50 (12.00, 24.50)	-0.794	0.427
Alkaline phosphatase (U/L)	98.70 ± 25.27	72.25 ± 18.00	3.312	0.003**
Urinary albumin (%)	3/7	2/18	1.92	0.300
Blood urea nitrogen (mmol/L)	6.66 (6.18, 10.65)	5.50 (3.97, 6.55)	-2.376	0.017*
Uric Acid (umol/L)	375.50 (141.75, 519.25)	304 (253, 391)	-0.311	0.755
Blood creatinine (mmol/L)	86.00 (62.75, 117.50)	68.7 (54.08, 74.95)	-1.716	0.086

Normally distributed data was compared using t-test. Non-normally distributed data was compared using the nonparametric Mann-Whitney test.

The rates between the two groups were compared using the chi-square test.

* = p < 0.05,

** = p < 0.01.

### Laboratory measurements

Patients with FT1DM had lower C-P0 and C-P2hr levels and higher Glu2hr, Glu0/C-P0, and Glu2hr/C-P2hr levels than those with T1DM (p < 0.05), suggesting that the regulation of glucagon secretion from α cells is impaired in FT1DM. GAD antibody levels in the FT1DM group were significantly lower than in the T1DM group (p < 0.05). There were no significant differences in HbA1c, INS-Ab, and Glu0 levels between the two groups (p > 0.05 for all) (Table 1).

Blood urea nitrogen (BUN) and alkaline phosphatase levels were significantly higher in the FT1DM group than in the T1DM group (p < 0.05). There were no significant differences in the blood routines (white blood cells, red blood cells, hemoglobin, and platelet count) or biochemical indexes (alanine aminotransferase, albumin, globulin, potassium, sodium, chlorine, cholesterol, triglyceride, high density lipoprotein, glutamine transpeptidase, blood creatinine, urinary albumin, and uric acid) between the two groups (p > 0.05 for all) (Table 1).

### CGMS results

The CGMS data showed that the percentage of blood glucose levels < 3.9 mmol/L was higher in the FT1DM group than in the T1DM group (p < 0.05). The Min blood glucose level in the FT1DM group was lower than the Min in the T1DM group (p < 0.05) (Table 2).

**Table 2:** CGMS data

Indexes	FT1DM (n = 10)	T1DM (n = 20)	95% confidence interval	t/z	P
Mean blood glucose (mmol/L)	8.86 ± 3.21	11.39 ± 3.36	(-5.1637, 0.0917)	-1.977	0.058
Standard deviation of blood glucose (mmol/L)	3.47 ± 1.07	3.12 ± 1.25	(-0.5912, 1.3012)	0.769	0.449	
Mean amplitude glycemic excursions (mmol/L)	9.56 ± 2.77	7.76 ± 3.72	(-0.9354, 4.5213)	1.346	0.189
Percentage of time above 13.9 (PT_1_)	8.00 (2.00, 49.00)	15.50 (1.50, 57.50)	/	-0.529	0.597	
AUC > 13.9 mmol/L	0.10 (0, 1.15)	0.325 (0, 2.05)	/	-0.880	0.379
Percentage of time below 3.9 (PT2)	16.00 (0, 34.50)	0 (0, 0)	/	-2.979	0.003**	
AUC < 3.9 mmol/L	0.10 (0, 0.20)	0 (0, 0)	/	-3.388	0.001**
The maximum (mmol/L)	16.16 ± 3.38	17.27 ± 4.40	(-4.3635, 2.1415)	-0.700	0.490
The minimum (mmol/L)	2.40 (2.20, 5.03)	5.15 (3.83, 6.55)	/	-2.360	0.018*

AUC = Area under curve. Normally distributed data was compared using t-test. Non-normally distributed data was compared using the nonparametric Mann-Whitney test. The rates between the two groups were compared using the chi-square test.

* = p < 0.05

** = p < 0.01.

Other CGMS parameters (MBG, SDBG, MAGE, Max and the percentage of blood glucose levels > 13.9 mmol/L) were similar between the two groups (p > 0.05 for all).

## DISCUSSION

In the present study, CGMS data showed that patients in the FT1DM group had a higher risk of hypoglycemia at the stable stage, worse destruction of pancreatic beta cells, and more severe dysfunction of the islet alpha cells than patients with T1DM. Renal dysfunction was more commonly found in patients with FT1DM. Other CGMS parameters did not differ significantly between the two groups.

There were no significant differences found in MBG, SDBG, MAGE and Max levels between the FT1DM and T1DM patients. These results indicate that blood glucose fluctuations were similar in the FT1DM and T1DM groups. However, the percentage of blood glucose levels < 3.9 mmol/L was higher and the Min blood glucose level was lower in the FT1DM group compared to the T1DM group (p < 0.05 for both). These findings suggest that patients with FT1DM have a higher risk of hypoglycemia compared to patients with T1DM. A 5-year follow-up study of 41 patients with FT1DM performed by Murase and cols. showed that blood glucose fluctuations (measured using 7-point blood glucose monitoring) and the frequencies of severe hypoglycemia were higher in a group of FT1DM patients than in a group of T1DM patients (9). The results of the current study partially agree with this, as we only found that patients with FT1DM had a higher risk of hypoglycemia compared to patients with T1DM, which was indicated by the higher percentage of blood glucose levels < 3.9 mmol/L and the lower minimum blood glucose levels in the FT1DM group. However, we did not find significant differences among other indexes for blood glucose fluctuations between the two groups. This difference may be associated with the methods that were used to monitor blood glucose levels. We used CGMSs to monitor blood glucose levels, which are more accurate and occur in real-time (10). We also noticed that our patients with FT1DM were older and had higher BMIs and waist-to-hip ratios than the T1DM patients. The finding of higher BMIs in patients with FT1DM compared with those with T1DM was consistent with results of previous studies (2,11-13). The higher BMIs in patients with FT1DM compared with those with T1DM may be due to the rapid onset and short duration of FT1DM, which does not reduce body weight significantly. However, beta cell function was severely damaged, which led to lower C-peptide levels in patients with FT1DM.

Furthermore, we observed that premeal doses of insulin (unit/kg/day) were significantly higher in the FT1DM group than in the T1DM group. However, while the total daily doses of insulin were higher in the FT1DM group, there was no significant difference between the two groups. One possible reason for the pre-meal doses of insulin may be related to the more severely damaged islet function in patients with FT1DM, i.e., the poor function of islet beta cells and the dysfunction of islet alpha cells, resulting in a need for more insulin to reduce the postprandial blood glucose level. Imagawa and cols. found that daily doses of insulin were significantly higher in 161 FT1DM patients than in T1DM patients in follow-ups at 3, 6, and 12 months (11). This is different from the results of our study, and this difference may be related to the sample size, as we only studied 10 patients whose total daily doses of insulin had a tendency to be higher (0.63 ± 0.20 u/kg/day) than those of T1DM patients (0.52 ± 0.18 u/kg/day, p = 0.109).

Islet function was more severely damaged in FT1DM patients, as they had lower C-P0 and C-P2hr levels and higher Glu2hr, Glu0/C-P0, and Glu2hr/C-P2hr levels compared with T1DM patients (p < 0.05). Though the pathogenesis of FT1DM is currently unclear, it may be mediated by a variety of factors: viral infections, pregnancy, drugs, etc., in addition to autoimmune and genetic factors that can trigger an accelerated immune reaction and promote massive beta-cell death in genetically susceptible individuals. In contrast, a small number of islet cells may still be viable in T1DM patients at the onset of the disease (2,6,14,15). Zheng and cols. proposed that the appearance of a low titer of autoantibodies was likely caused by the viral infection destroying the islet cells (3). Shibasaki and cols. suggested that FT1DM is associated with viral infections and is affected by the patient's genetic background (16). However, Imagawa and cols. found that although GAD-AB, INS-AB, and other related antibodies appeared in patients with FT1DM, they were in low antibody titers and appeared in a short-term national survey (11). Studies have suggested that intestinal viruses and chemokines not only damage the islet beta cells but also accelerate the autoimmune reaction, which destroys the remaining islet beta cells (17,18). Our study found that the GAD-AB was significantly lower in the FT1DM group than in the T1DM group, while the INS-AB titer findings were similar between the two groups. This is consistent with previous reports (3,9).

Patients with FT1DM had lower C-P0 and C-P2hr levels than patients with T1DM in our study, which could be related to the stage of onset of disease (2,6). This suggests that the function of islet beta cells in FT1DM was rapidly damaged from the onset of the disease, progressively declined, and could still be declining in T1DM after a few years. Meanwhile, patients with FT1DM had higher Glu2hr, Glu0/C-P0, and Glu2hr/C-P2hr levels than those with T1DM, suggesting that glucagon secretion was also damaged in the FT1DM group compared to the T1DM group.

The results of our study were consistent with those of the studies of Liu and Fany (5,19), who more clearly showed a regulating function disorder in the islet alpha cells in an FT1DM group. Sayama and cols. showed through morphological observations that islet alpha and beta cells were destroyed in FT1DM patients, while islet beta cells were destroyed and islet alpha cells remained functional in T1DM patients at the early stage of the disease (20). Fan and cols. followed 6 FT1DM patients for 9-72 months and reported that fasting and postprandial C-P2hr levels were close to levels requiring hospitalization at the acute stage of the disease, which suggested that the islet beta cells were completely and irreversibly destroyed (19). A report by Huang and cols. showed that the function of the islet beta cells in 2 FT1DM patients did not improve after 7 months of follow up (21).

There were no significant differences in white blood cells, neutrophils, alanine, aspartate, potassium, cholesterol, high density lipoprotein, creatinine, and glutamine transpeptidase levels between the two groups. However, cholesterol and high-density lipoprotein levels were lower and white blood cells, neutrophils, alanine, aspartate, potassium, creatinine, and glutamine transpeptidase levels were generally higher in FT1DM patients at the acute stage [2]. This suggests that FT1DM caused not only metabolic and biochemical disorders but also tissue dysfunction or injuries, including in the liver and kidney, at the acute stage. Though metabolic and some biochemical indicators could return to the normal range after treatment at the stable stage, the liver and kidney injuries are irreversible, as indicated by the BUN and alkaline phosphatase levels being significantly higher in the FT1DM group compared to the T1DM group (P < 0.05). BUN is an important indicator of renal function, while alkaline phosphatase is mainly derived from the liver, bone, and kidneys; it indicates early kidney damage (22-24). Compromised kidney function following the acute stage of FT1DM may be caused by large blood glucose fluctuations, immune reactions, inflammation, and severe hypoglycemia.

In conclusion, the present study suggests that patients with FT1DM at the stable stage and patients with T1DM had similar blood glucose fluctuation ranges. However, patients with FT1DM had a higher proportion of hypoglycemic levels, severely damaged pancreatic beta cells and more severe dysfunction of the islet alpha cells and kidney compared to patients with T1DM.
